# Differential trends in the rising incidence of endometrial cancer by type: data from a UK population-based registry from 1994 to 2006

**DOI:** 10.1038/bjc.2011.68

**Published:** 2011-04-26

**Authors:** T Evans, O Sany, P Pearmain, R Ganesan, A Blann, S Sundar

**Affiliations:** 1 West Midlands Cancer Intelligence Unit, University of Birmingham, Birmingham B15 2TT, UK; 2 Pan Birmingham Gynaecological Cancer Centre, City Hospital, Dudley Road, Birmingham B18 7QH, UK; 3 Department of Pathology, Birmingham Women's Hospital, Birmingham B15 2TG, UK; 4 Department of Medicine, City Hospital, Dudley Road, Birmingham B18 7QH, UK; 5 School of Cancer Sciences, University of Birmingham, Edgbaston, Birmingham B15 2TT, UK

**Keywords:** endometrial cancer, histology, incidence, type 1, type 2, deprivation

## Abstract

**Background:**

Endometrial cancer is the most common gynaecological cancer in the western world, the incidence increasing in the United Kingdom by over 40% since 1993. Two types of endometrial cancer exist – oestrogen-dependent type 1 with good prognosis and non-oestrogen-dependent type 2 with poor prognosis. The histopathological distribution of the increase in endometrial cancer is unknown. This study investigates the observed incidence trends of the two types, the age, stage, and socioeconomic distribution of this increase and survival outcome.

**Methods:**

Data were analysed from 6867 women with endometrial cancer registered between 1994 and 2006, at a UK population-based cancer registry.

**Results:**

Increased endometrial cancer incidence is confined to type 1 cancers with a significant increase in age standardised incidence rate (ASR) from 12.0 per 100 000 (confidence interval (CI) 10.7–13.2) in 1994 to 16.3 per 100 000 (CI 14.9–17.7), *P*<0.001 in 2006, while ASR of type 2 cancer changed from 2.5 per 100 000 (CI 2.0–3.1) in 1994 to 2.2 per 100 000 (CI 1.7–2.7) in 2006, which was not statistically significant *P*>0.05. Increase in type 1 cancer is most marked in age groups 60–69 years (*P*<0.001) and 70–79 years (*P*<0.001) and distributed equally among socioeconomic quintiles. While outcome for type 1 cancer has improved, 1-year survival in type 2 cancer is unchanged from 73.1% in 1994 to 74.3%, *P*=0.089 and 5-year survival decreased from 55.1% to 40.9%, *P*=0.001.

**Conclusion:**

Increased incidence in endometrial cancer is confined to type 1 cancers, seen most in the 60–79 age groups and across all socioeconomic quintiles. Survival in type 2 cancer has decreased significantly. Urgent research is needed to investigate prevention strategies in type 1 and improve therapy in type 2 cancers.

Endometrial cancer is now the most common cancer of the female genital tract in the western world, with an estimated worldwide incidence of 287 000 and 74 000 deaths from endometrial cancer in 2008 (Ferlay *et al*, 2010). Endometrial cancer has increased by over 40% in the United Kingdom since 1993, to 7536 cases in 2007 and 1741 deaths in 2008 (http://info.cancerresearchuk.org/cancerstats/ types/uterus/). While the incidence and mortality rates of several other cancers have plateaued or decreased in the last decade the incidence of endometrial cancer has been rising throughout Europe (Jemal *et al*, 2008). This increase has been attributed to increasing obesity, life expectancy, and adjuvant Tamoxifen use for breast cancer (Bray *et al*, 2005).

Endometrial cancer can be classified into two distinct groups – type 1 and type 2 based on histology, which differ in molecular as well as in clinical and histopathology profiles (Bokhman, 1983). Type 1 endometrial cancer is oestrogen-driven, of endometrioid histology, arises in the background of endometrial hyperplasia, is strongly linked to obesity, occurs predominantly in pre and perimenopausal women and is associated with good prognosis (>90% 5-year survival rate) (Doll *et al*, 2008). Type 2 is non-oestrogen dependent, non-endometrioid, with higher grade histologies, specifically uterine papillary serous carcinomas (UPSCs) and clear-cell carcinomas, more aggressive, and carries an adverse prognosis (Amant *et al*, 2006; Mendivil *et al*, 2009). Although type 2 cancers contribute only 10% of endometrial cancer incidence, they cause 50% of recurrence and deaths from endometrial cancer with a low 5-year, all stage, overall survival rate of 35% (Singh *et al*, 2008; Bansal *et al*, 2009). Type 2 cancers typically arise in an atrophic endometrial background, and often have deep myometrial penetration and demonstrate lymph node spread. They usually occur at an older age, ∼5–10 years later than type I tumours. In addition, type 1 endometrial carcinomas are characterised by mutations in PTEN, PIK3CA, KRAS, and *β*-catenin, along with microsatellite instability; type 2 endometrial carcinomas are characterised by genetic alterations in p53, HER-2/neu, p16, and E-cadherin (Llobet *et al*, 2009).

Although the increase in incidence in endometrial cancer is well recognised, the distribution of this increase across the two types of endometrial cancer is not known. This information has vital implications for health policy and the development of targeted therapies in endometrial cancer. Understanding the trends in incidence of endometrial cancer and socioeconomic distributions may help inform strategies to improve early diagnosis and therapy and target healthcare resource efficiently.

In this study, we present an analysis of the observed incidence trends of the two major types of endometrial cancer in the West Midlands area of the United Kingdom from 1994 to 2006. We sought evidence of a differential increase between type 1 and type 2 cancers over this time period, and whether or not this varied with socioeconomic distribution. We also explored age and stage distribution of endometrial cancers over this time period and survival outcome based on histology type and socioeconomic deprivation.

## Methods

### Population details

Analysis was performed of prospective data collated on all newly diagnosed endometrial cancer patients registered between 1994 and 2006 at the West Midlands Cancer Intelligence Unit (WMCIU). Registration of a cancer diagnosis usually occurs within 3 months of diagnosis, though registration of all clinical information can take longer. All analysis involving patient identifiable data was conducted on fully complete cases in house by the researchers employed by the WMCIU who are authorized to access these confidential data. The data were then tabulated for use within the paper. The WMCIU houses a population-based cancer registry serving the 5.4 million residents in the West Midlands region of the United Kingdom. All neoplasms of invasive, *in situ*, uncertain or unknown behaviour are recorded on the WMCIU's central database. The WMCIU aims to register each case from a range of data sources to ensure the most accurate information possible – full coding of a cancer case may involve data from hospital patient information systems, pathology reports, medical records departments, radiotherapy systems, hospices, other cancer registries, general practices, private hospitals, cancer screening programmes, nursing homes, and death certificates.

### Histology

The registry records data on the histology of the cancer as determined by the pathologist issuing the histology report at the time of diagnosis of cancer. These histology reports were categorised into the International Statistical Classification of Diseases and Related Health Problems, 10th Edition, Volume 1 (World Health Organisation) (ICD-10) code C54=malignant neoplasm of corpus uteri and code C55=malignant neoplasm of uterus, part unspecified. Specific histopathological tumour types were classified in accordance with the International Statistical Classification of Diseases and Related Health Problems, 10th Edition, Volume 3 (World Health Organisation), referring to the morphology of the cancer site by the same international classification. These morphologies were then further grouped into type 1 and type 2 endometrial cancers by the specialist histopathologist in gynaecological cancer on the team (RG). The WMCIU researchers then applied the appropriate type, based on the groupings produced, to the actual cases included within the study so that the analysis could be carried out.

Type 1 included common indolent types of endometrial adenocarcinoma, which were coded as grades 1–3 endometrioid, mucinous, or adenocarcinoma. Type 2 encompassed the more uncommon and highly aggressive types of uterine cancer. These included those coded as serous, clear-cell, carcinosarcoma or malignant mixed mullerian tumour. Uterine sarcomas and other types of endometrial cancers that are distinct from the above two subtypes were excluded from analysis (Table 1).

### Social deprivation

The Indices of Multiple Deprivation 2004 (IMD 2004) is a deprivation index at the small area level, created by the British Department for Communities and Local Government. Commisisoned and published by the Department for Communities and Local Government, the Indices of Deprivation 2004 are based upon a mix of 2001 national census data and 2001 administrative data sources. These have been updated with more recent (2005) administrative data sources from Government Departments and have been published as Indices of Deprivation 2007 (Department for Communities and Local Government TEIoDoL). IMD 2004 is made up of seven distinct dimensions of deprivation called domain indices, which relate to deprivation in income, employment, health and disability, education skills and training deprivation, barriers to housing and services, living environment deprivation, and crime. The deprivation score of the area where the individual lives is assumed to be characteristic of the deprivation experienced by all individuals living there. Scores are calculated at small geographical areas (lower super output areas (LSOA), each containing ∼1500 people), but there may still be some variation in deprivation within each LSOA which is not captured in this analysis. All LSOAs in England were ranked according to their deprivation (measured by the 2004 income domain score) and divided into five equal groups (quintiles). The top 20% was defined as the ‘most affluent population of England’. All LSOAs in the West Midlands that were within the scores for the ‘most affluent population of England’ were defined as ‘the most affluent population’ in the West Midlands, and conversely LSOAs that were within the scores for ‘the least affluent population of England’ were defined as the ‘least affluent population’ in the West Midlands. Each cancer registration is routinely assigned a deprivation score and quintile by the WMCIU based upon the diagnosis postcode/address information received for each case. The data for this study therefore already contain the deprivation index. Because of the possibility of ‘mathematical coupling’ whereby the relationship between IMD 2004 and markers of health can be predicated by including the health domain of IMD 2004, this analysis focuses only on the income domain of IMD 2004 where 1=the most deprived and 5=the least deprived (Archie, 1981; SIGN, 2002).

### Statistical analysis

Age standardised incidence rates (ASRs) were calculated via the direct method. Age standardisation was conducted using the standard European population throughout; all figures are based on a female population. To assess the statistical significance of the results, 95% confidence intervals (CIs) were also calculated for the ASRs using the Poisson distribution (Breslow NEaDNE, 1987). Relative survival analysis was performed to calculate survival rates. Relative survival is defined as the observed survival in the patient group divided by the expected survival of the general population, matched by age, sex, and calendar year (Dickman *et al*, 2004). Relative survival was calculated in STATA (v.11) (StataCorp LP, College Station, TX, USA) using the strs programme which calculates relative survival estimates using the Ederer II method (Evaluation Section NCI, 1959; http://www.pauldickman.com/survival/strs.pdf). National life tables were obtained from the Cancer Research UK Cancer Survival Group at the London School of Hygiene and Tropical Medicine. One- and five-year relative survival was calculated using 5 year rolling averages.

The incidence of endometrial cancer, its two subtypes, and the relative proportions were tested with Pearson's correlation with year of diagnosis for all subjects. For analysis of incidence by socioeconomic class, we pooled data from classes 1 and 2, and classes 4 and 5 for improved power. This therefore gives two groups of approximately equal sample size. We did not analyse data from class 3 as it lacks the power for a rigorous analysis. *P*-value of 0.05 was considered significant.

## Results

A total of 7465 women with endometrial cancer were registered at the WMCIU during 1994–2006. We excluded 598 cases that did not conform to either type 1 or type 2 histology, giving a final total of 6867 cases. Of these, 5903 were classified as type 1 and 964 as type 2 endometrial cancers as per histology classified in Table 1. The proportion of excluded morphologies remained fairly constant over time, with, on average 8% excluded between 1994 and 2006 (Table 1). Less than 2% of endometrial cancers analysed as type 1 or type 2 and utilised for this analysis were death certificate only (DCO) registrations. These cases have been included for the purpose of calculating incidence rates as they represent real cases and so trends in incidence of these endometrial cancers were analysed. Given that DCOs represent <2% of cases, they have been removed from the survival analysis due to insufficient information regarding the duration of disease and the possible bias this could introduce to the analysis. This is a widely used approach applied in other studies of cancer survival (De Angelis *et al*, 2009; Coleman *et al*, 2011).

**Figure 1:**
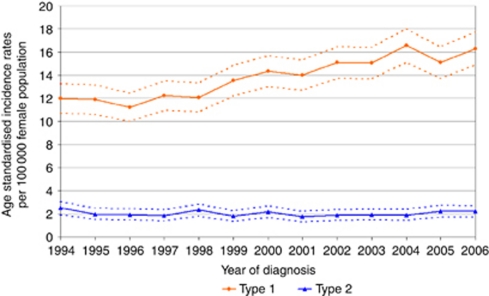
Differential trends in endometrial cancer incidence across the two types. Age standardised incidence rates and confidence intervals are shown.

An overall increase in the ASR of endometrial cancer was observed over the time period, from 16.1 per 100 000 population (CI 14.6–17.5) in 1994 to 19.6 per 100 000 population (CI 18.1–21.1) in 2006. Analysis by type of endometrial cancer revealed a marked differential trend in incidence by type. The ASRs of type 1 cancers showed a highly significant linear increase from 12.0 per 100 000 (CI 10.7–13.2) in 1994 to 16.3 (CI 14.9–17.7) in 2006, *r*=0.94, *P*<0.001. However, the ASR of type 2 cancers remained static; 2.5 per 100 000 (CI 2.0–3.1) in 1993 compared with 2.2 per 100 000 (CI 1.7–2.7) in 2006, *r*=−0.15, *P*-value=0.633. This is represented graphically in Figure 1.

**Figure 2:**
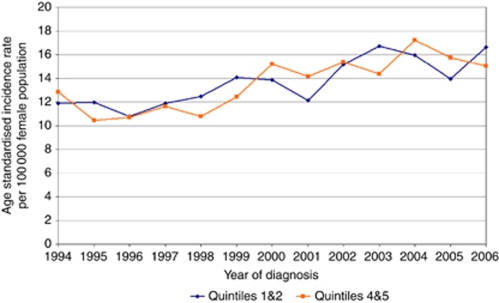
Analysis of increase in type 1 endometrial cancer rates by socioeconomic distribution. Confidence intervals for both graphs overlap at each time point.

**Figure 3:**
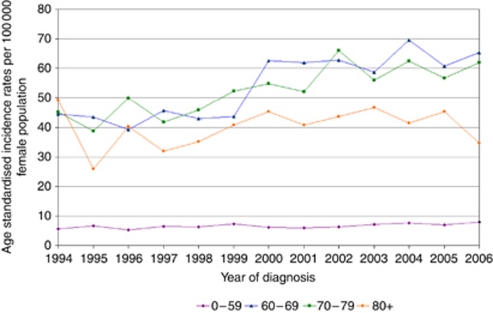
Analysis of type 1 endometrial cancer incidence by age groups.

The overall distribution of our cases among the socioeconomic quintiles was roughly similar with the exception of the smaller proportion of cases overall that were diagnosed in the most affluent quintile (Table 2; quintiles 1–5 with 1 being most deprived and 5 being least deprived). Analysis of the increase in type 1 endometrial cancer by socioeconomic distribution revealed that this increase in incidence rates was evenly distributed among all socioeconomic quintiles. There was a strong relationship between the rates of increase in groups 1 and 2 *vs* groups 4 and 5, *r*=0.74, *P*=0.004. Figure 2 represents an analysis of type 1 cancer incidence comparing groups 1 and 2 with groups 4 and 5. When analysing the distribution of all cases by age, the largest proportion of cases fell in the 60–69 age group, which accounted for 31% of all cases. The 0–59 group was the second largest, although in over 99% of cases, the age at diagnosis was in the 30–59 age group (Table 3). To investigate the impact of increased life expectancy on type 1 cancer we analysed the ASRs in age groups 0–59, 60–69, 70–79, and over 80 years. An increase in type 1 endometrial cancer was seen in age groups, 0–59 years: *r*=0.707, *P*=0.007; 60–69 years: *r*=0.861, *P*<0.001; and 70–79 years: *r*=0.838, *P*<0.001 over the study period, while the age standardised incidence in the over 80 years remained static; 80+ years: *r*=0.24, *P*=0.418 (Figure 3).

**Figure 4:**
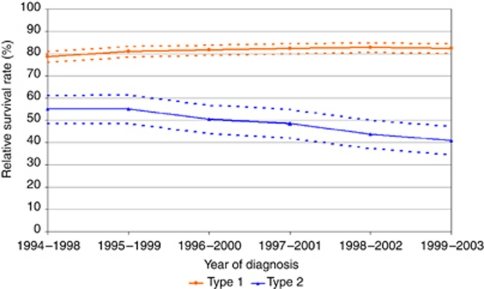
Five-year relative survival of endometrial cancer types 1 and 2.

Over the study period, there was an overall improvement in 1-year and 5-year relative survival rates for all morphologies combined (types 1 and 2 and all other morphologies) of endometrial cancer. One-year survival improved from 85.6% in time period 1994–1998 to 90.3% by 2003–2007, and 5-year survival from 73.2% in 1994–1998 to 75.8% in 1999–2003, *r*=0.86, *P*=0.001. This improvement was limited to type 1 cancer, with 1-year survival improving from 89.1% in 1994–1998 to 94.7% by 2003–2007, *r*=0.94, *P*<0.001. Five-year survival in type 1 cancer increased slightly from 78.7% to 82.4%, *r*=0.33, *P*=0.950, but the difference was not statistically significant. Specifically, outcome for type 2 cancers demonstrated a deterioration over time, with 1-year relative survival unchanged from 73.1% in 1994 to 74.3%, *r*=−0.56, *P*=0.089 and 5-year relative survival decreasing from 55.1% to 40.9%, *r*=−0.98, *P*=0.001 over the study period (Figure 4).

We investigated survival by individual deprivation quintile and by separate subtype, however, due to small numbers, the data were found to lack robustness and so have not been presented. To investigate the deprivation gap in relative survival for endometrial cancer we therefore combined all morphologies (type 1, type 2 and all other morphologies). One-year survival rates for endometrial cancer showed no impact as a result of deprivation, 2003–2007 data, groups 1 and 2 showed 90.5% (CI 88.4–92.2%) while groups 4 and 5 showed 90.0% (CI 87.9–91.7%) relative survival rates. However, analysis of 5-year survival in women diagnosed from time periods 1994 to 2003, demonstrated a significant improvement in relative survival in more affluent groups 4–5, from 74.8% to 78.5%, *r*=0.66, *P*=0.036, while survival was not significantly better, 69.8–72.4%, *r*=0.66, *P*=0.151 in less affluent groups 1–2, over the study period. The difference in survival between groups 1–2 and 4–5 was not significant, *r*=0.27, *P*=0.6. Type 1 cancer was more frequently diagnosed at an earlier stage, 69% at stages 1 and 2 *vs* 43% at stages 1 and 2 for type 2 cancers. However, these data have to be interpreted with caution, as data on stage were incomplete in at least 20% of cases.

## Discussion

This study identifies a differential increase in endometrial cancer from 1994 to 2006 based on data from a population-based cancer registry, with a marked increase in oestrogen-dependent type 1 cancers and a static level of type 2 cancers. To our knowledge, this is the first paper to demonstrate this differential increase. Investigation of ASRs in the different age groups revealed an increase across most age groups 0–79 years, most markedly in age groups 60–69 and 70–79 years. Our data demonstrate the likelihood of an enduring type 1 endometrial cancer risk from hyper-oestrogenic states that persists well beyond the perimenopausal time frame. There is a need for research in prevention strategies that can target this persistent risk, for example, long-term prophylaxis with progesterone or the Mirena progesterone coil, particularly in those most at risk.

Increasing body mass index shows a strong linear relationship with endometrial cancer and some postulate that the incidence of endometrial cancer will rise to twice the 2005 rates by 2015 (Bjorge *et al*, 2007; Reeves *et al*, 2007; Renehan *et al*, 2008; Lindemann *et al*, 2008, 2010). In the affluent developed world, obesity is unevenly distributed with greater prevalence in the more deprived socioeconomic groups (Friel *et al*, 2007). The prevalence of obesity in women has been shown to rise steadily and significantly with increasing area deprivation from 20.1% in the least deprived to 33.1% in the most deprived (http://www.scotpho.org.uk/home/Clinicalriskfactors/ Obesity/obesity_data/obesity_deprivation.asp). Given that obesity is a significant main driver for oestrogen-related endometrial cancer incidence, we investigated if a difference in incidence within socioeconomic quintiles could be shown. Interestingly, we did not find any difference in distribution among the quintiles with no difference between quintiles 1–2 and quintiles 4–5 and this has remained so over the 12 years in the study period. Our findings are consistent with national data in endometrial cancer and suggest that the relationship between endometrial cancer incidence, obesity, and deprivation is complex (Cooper *et al*, 2008).

The significant deterioration in 5-year survival rates for type 2 cancers, while incidence remains static, may reflect greater awareness of pathologists of these histologies and improved pathological classification. Our findings are consistent with the Surveillance, Epidemiology and End Results database (SEER), United States study of >45 000 women with endometrial cancer, which suggest that there is an increase in mortality which may be related to advanced-stage cancers and high-risk histologies namely UPSC and clear-cell carcinoma (Ueda *et al*, 2008). A significant deprivation gap in survival exists between the most affluent and the most deprived groups with endometrial cancer of ∼4% based on data from England and Wales with survival lagging 2–6% behind Europe (Kitchener, 2008). Our study confirms that recent improvements in 5-year survival are seen mostly in the more affluent groups, showing that more work is needed to overcome this discrepancy.

The strengths of the study are that it is population based, with data subjected to robust quality assurance and, and is therefore likely to accurately reflect changes in incidence in a broader population. This methodology of using a high resolution look at population-based cancer registry data captures trends and significant findings and has been validated previously (Gatta *et al*, 2010). Some caveats with this study's findings must be noted. The West Midlands is a diverse region of the United Kingdom, comprising 9% of the population of the United Kingdom with wide variation between urban inner city areas and large rural areas. The region has a higher multiethnic population, 11.3% comprising ethnic minorities. The largest ethnic minority community is Indian (3.4%), followed by Pakistani (2.9%) and Black Caribbean (1.9%) (http://www.statistics.gov.uk/census2001/profiles/ commentaries/west_midlands.asp#Population, National Statistics, 2001). This population profile may impact on our results. We were unable to investigate ethnicity data and relationship with endometrial incidence, as the classification of ethnicity changed during the study period preventing robust analysis. However, given that the mortality and age profiles of the West Midlands are similar to the rest of the United Kingdom, we believe the results are valid and are likely to reflect profiles of endometrial cancer in the broader population. Because of small numbers, we were also unable to assess any differences in survival for type 1 and type 2 endometrial cancers by socioeconomic group. With data from only one cancer registry, the confidence intervals are quite large and repeating this high resolution analysis with data from a number of registries may yield greater insight and enable further analysis. While some cases of type 2 endometrial cancer specimens during 1994–2006 were sent to the regional gynaecological cancer centre for central pathology review by a histopathologist specialising in gynaecological cancers this was not uniform and a further study with expert review of pathology may add greater insight. However, given that the percentage of type 2 cancers has remained static over the time period, we believe that the influence of expert pathology on classification of these tumours is unlikely to impact significantly on the incidence trend observed. Since the study period, there has been greater centralisation of gynaecological oncology services and the impact of this on survival will need to be assessed in future studies.

## Conclusion

Our study shows that the increase in incidence in endometrial cancer is confined to type 1 endometrial cancer, while type 2 cancer has remained static over the 12-year study period. This increase is seen across all deprivation quintiles and all age groups except the over 80 years. The most significant increases are seen in the 60–79 age groups, suggesting that the oestrogenic impetus for developing type 1 endometrial cancer extends well beyond the perimenopausal years. Survival in type 2 cancers remains poor and has deteriorated. Urgent research into prevention strategies for type 1 endometrial cancer and the role of targeted therapies in type 2 cancer is needed. Research is also needed to improve early diagnosis by public health education and better referral pathways. These may improve outcome in the more deprived groups.

## Figures and Tables

**Table 1:** Classification of endometrial cancer into types 1 and 2 and histology excluded from analysis

**Type 1**	**Type 2**	**Excluded histologies**
Adenocarcinoma NOS (ICD10, C54 & C55)	Adenocarcinoma with neuroendocrine differentiation (ICD10, C54)	Adenocarcinoma with mixed subtypes (ICD10, C54)
Adenosquamous carcinoma (ICD10, C54 & C55)	Carcinoma, anaplastic NOS (ICD10, C54 & C55)	Adenocarcinoma with squamous metaplasia (ICD10, C54 & C55)
Carcinoma NOS (ICD10, C54 & C55)	Carcinoma, undifferentiated NOS (ICD10, C54 & C55)	Adenocarcinoma, metastatic NOS (ICD10, C54 & C55)
Endometrioid adenocarcinoma (ICD10, C54 & C55)	Carcinosarcoma NOS (ICD10, C54 & C55)	Adenoid SCC (ICD10, C55)
Endometrioid adenocarcinoma, ciliated cell variant (ICD10, C54)	Clear-cell adenocarcinoma NOS (ICD10, C54 & C55)	Acantholytic SCC (ICD10, C55)
Mucin producing adenocarcinoma (ICD10, C54)	Mesodermal mixed tumour (ICD10, C54 & C55)	Adenosarcoma (ICD10, C54 & C55)
Mucinous, mucoid adenocarcinoma (ICD10, C54)	Mullerian mixed tumour (ICD10, C54 & C55)	Carcinoma, metastatic NOS (ICD10, C54 & C55)
	Papillary adenocarcinoma NOS (ICD10, C54)	Choriocarcinoma NOS (ICD10, C55)
	Papillary carcinoma NOS (ICD10, C54)	Endometrial stromal sarcoma (ICD10, C54 & C55)
	Papillary cystadenocarcinoma NOS (ICD10, C54)	Epithelioid leiomyosarcoma (ICD10, C54 & C55)
	Papillary serous cystadenocarcinoma (ICD10, C54)	Leiomyosarcoma NOS (ICD10, C54 & C55)
	Serous cystadenocarcinoma NOS (ICD10, C54 & C55)	Mesenchymoma, malignant (ICD10, C55)
	Serous surface papillary carcinoma (ICD10, C54)	Mixed cell adenocarcinoma (ICD10, C54)
		Neoplasm, malignant (ICD10, C54 & C55)
		Neoplasm, metastatic (ICD10, C55)
		Pseudoglandular SCC (ICD10, C55)
		Rhabdomyosarcoma NOS (ICD10, C54)
		Sarcoma NOS (ICD10, C54 & C55)
		Sarcomatosis NOS (ICD10, C55)
		Signet ring cell carcinoma (ICD10, C54)
		Solid carcinoma NOS (ICD10, C55)
		Spindle cell carcinoma (ICD10, C54 & C55)
		Spindle cell sarcoma (ICD10, C55)
		Squamous cell carcinoma NOS (ICD10, C54 & C55)
		Stromal sarcoma, NOS (ICD10, C54 & C55)
		Teratoma, malignant NOS (ICD10, C54)
		Transitional cell carcinoma NOS (ICD10, C54)
		Tumour cells, malignant (ICD10, C54)

Abbreviations: ICD=International Classification of Disease; NOS=not otherwise specified; SCC=squamous cell carcinoma. Note that this table only accounts for the tumours registered on the West Midlands Cancer Intelligence Unit (WMCIU) database, therefore where morphologies have only been assigned to a single ICD10 cancer code, this reflects the WMCIU database and does not necessarily mean that morphology is only diagnosed in that ICD10 cancer site.

**Table 2:** Socioeconomic distribution of endometrial cancers diagnosed in the West Midlands, 1994–2006

**Deprivation quintile**	**Proportion (%)**
Least affluent	21.1
Less affluent	20.7
Average	20.4
More affluent	22.6
Most affluent	15.1

**Table 3:** Age distribution of endometrial cancers diagnosed in the West Midlands, 1994–2006

**Age group (years)**	**Proportion (%)**
0–29	0.1
30–34	0.3
35–39	0.9
40–44	1.7
45–49	3.8
50–54	8.8
55–59	14.2
60–64	15.2
65–69	15.3
70–74	13.5
75–79	12.0
80–84	7.7
85+	6.3
